# GreedyPlus: An Algorithm for the Alignment of Interface Interaction Networks

**DOI:** 10.1038/srep12074

**Published:** 2015-07-13

**Authors:** Brian Law, Gary D. Bader

**Affiliations:** 1 Department of Computer Science, University of Toronto, Toronto, ON, Canada; 2 The Donnelly Centre, University of Toronto, Toronto, ON, Canada; 3 Department of Molecular Genetics, University of Toronto, Toronto, ON, Canada

## Abstract

The increasing ease and accuracy of protein-protein interaction detection has resulted in the ability to map the interactomes of multiple species. We now have an opportunity to compare species to better understand how interactomes evolve. As DNA and protein sequence alignment algorithms were required for comparative genomics, network alignment algorithms are required for comparative interactomics. A number of network alignment methods have been developed for protein-protein interaction networks, where proteins are represented as vertices linked by edges if they interact. Recently, protein interactions have been mapped at the level of amino acid positions, which can be represented as an interface-interaction network (IIN), where vertices represent binding sites, such as protein domains and short sequence motifs. However, current algorithms are not designed to align these networks and generally fail to do so in practice. We present a greedy algorithm, GreedyPlus, for IIN alignment, combining data from diverse sources, including network, protein and binding site properties, to identify putative orthologous relationships between interfaces in available worm and yeast data. GreedyPlus is fast and simple, allowing for easy customization of behaviour, yet still capable of generating biologically meaningful network alignments.

A major objective of biology is to understand how complex biological systems are assembled from their components into functional units and how they evolve. In molecular biology, efforts have increasingly focused on how proteins and other molecules interact, and determining how their interplay affects biological phenotypes, including disease. This has driven work in interactomics, as better, cheaper high-throughput methodologies allow us to systematically map the dynamic molecular interactions in a cell^1^. To aid the evolutionary study of these networks, a number of network alignment methods have been developed^2^.

Recently, protein interactions have been mapped at the level of amino acid positions, which can be represented as an interface-interaction network (IIN), where vertices represent binding sites, such as protein domains and short sequence motifs^3^,^4^,^5^,^6^,^7^. These networks provide a more accurate picture of how protein interaction networks are organized in biological systems. Thus, studying the function and evolution of these higher resolution networks should provide new biological insights. However, current protein interaction network alignment algorithms are not designed to align these networks and generally fail to do so in practice. In response, we developed GreedyPlus, the first algorithm designed to align IINs. In the next sections, we provide background about the network alignment problem, introduce IINs and review existing algorithms. We then describe the GreedyPlus algorithm and associated input data, comparisons with existing protein-protein interaction network alignment methods and results aligning IINs from different species.

## Network Alignment Theory

In the trivial case of finding the ideal alignment of a network to itself, the network alignment problem is equivalent to the classic graph isomorphism problem, which is of unknown complexity^8^. However, as biological networks evolve, we expect divergence between the networks via the addition and deletion of both vertices and edges, and thus the objective of network alignment is to find similarity between networks rather than perfect isomorphisms. In the particular case where one network is a subnetwork of the other, the network alignment problem is specifically the subgraph isomorphism problem^9^. In the general case, the network alignment problem degenerates into many instances of the subgraph isomorphism problem with loosened constraints; particularly, the objective is to find a set of non-overlapping, partial isomorphisms of all possible subnetworks of both networks. Given that the complete protein-protein interaction networks (PPINs) of species such as human and yeast^10^,^11^ number in the thousands of vertices and edges, and that the subgraph isomorphism problem is NP-complete, an optimal computational approach is unfeasible and heuristics and approximations must be used.

### Interface-Interaction Networks

Interface-interaction networks (IINs) are a refinement of PPINs wherein proteins are subdivided into their separate interaction interfaces^3^. We choose to represent the IIN using a traditional graph model where a vertex represents a specific binding site and an edge represents a physical interaction between two binding sites on their respective proteins. The IIN is thus a higher resolution version of the PPIN.

The higher resolution of IINs allows for new biological insights that cannot be derived from standard PPINs. For example, IINs can distinguish between “date hubs” – proteins that interact with many partners, but at different times or in different locations - and “party hubs” – proteins that interact with many partners simultaneously^12^. While these distinct types of hub proteins will appear identically in a PPIN, in an IIN, the former will have few binding sites that are reused for many different interaction partners whereas the latter will have many binding sites that are specific for each interaction partner. This is useful to help elucidate the evolutionary processes and constraints acting on hub proteins. The study of IINs will also help interpret how domain and binding site gain and loss affect the PPIN, predict PPIN perturbations caused by sequence mutations that affect binding sites, and allow in-depth analysis of how protein-protein interactions are formed and lost^5^,^13^,^14^.

Topological differences between IINs and PPINs, however, mean that algorithms designed to operate on PPINs may not function properly with IINs. While PPINs are often sparse, IINs are much more so, with each PPIN vertex (protein) split into multiple vertices that represent the different binding sites on that protein. Similarly, while PPINs exhibit a hub and spoke topology, with many low-degree and fewer high-degree vertices, this characteristic is exaggerated in IINs. For example, protein-recognition modules, such as protein kinases or SH3 domains, are often capable of binding many different proteins, leading to relatively few high-degree vertices connected to many low degree vertices. Additionally, due to binding specificity similarities, different domains will often recognize the same ligands, forming a multi-fan topology. Methods that depend on the neighbourhoods of vertices being topologically distinct to generate their alignments get confused by these repeated patterns and thus perform inconsistently (see below for examples).

Experimentally mapped interface-interaction data across species have recently become available, such as a set of interactions mediated by SH3 domains in *Saccharomyces cerevisiae* (budding yeast)^4^ and *Caenorhabditis elegans* (worm)^5^ SH3 domains are peptide-recognition modules that bind to short linear peptides with characteristic proline-rich motifs. The resulting IINs are bipartite, though this may not be generally the case. Due to their bipartite property, certain topological motifs, such as cliques, are absent while others, such as 4-cycles, are highly enriched. Existing PPIN alignment algorithms have not been designed for bipartite networks and can fail to align these networks. The graphlet degree signature similarity measure used by GRAAL^15^,^16^, for example, loses most of its resolution on a bipartite graph due to the absence of odd cycles. Alternatively, the bipartite nature of the networks confounds IsoRank^17^, as its vertex similarity measure can get stuck oscillating between domain and binding site vertices rather than converging.

To address the IIN alignment problem, we developed a new algorithm called GreedyPlus, which considers bipartite IINs by design.

### Protein-Protein Interaction Network Alignment

Even though we argue that IIN alignment represents a different problem to PPIN alignment, the problems are related in their approach and we review PPIN alignment work here. Previous network alignment research has focused on protein-protein interaction networks, although other network types have been studied^18^. Previous PPIN alignment methods have sought to identify pairs of orthologous proteins and/or functionally orthologous proteins. Mirroring biological sequence alignment techniques, PPIN alignment methods have broadly taken two approaches: local alignment and global alignment. Local alignment algorithms seek small subnetworks that are topologically similar, emphasizing regions of high-confidence alignment between the two networks. Typically, these methods use protein sequence alignment as a primary indicator of protein orthology, and then incorporate network information to identify clusters of sequence-similar proteins; these clusters in the network, then, are considered putatively orthologous functional units.

PathBLAST^19^, one of the first published PPIN alignment methods, and its successor NetworkBlast^20^, are examples of local network alignment algorithms. Both methods begin by identifying all pairs of proteins between the two input networks with significant sequence similarity (using BLAST e-values)^21^, formulating each pair as nodes within a global alignment graph, and filling in the edges between these paired protein nodes using interaction data. In the global alignment graph, edges can be aligned (edges exist in both input networks), gapped (an edge exists in only one input network), or mismatched (no edge exists in either network), implying an abstract model of network evolution. A scoring model is then used to score the aligned proteins, and the high-scoring pairings are combined into a small pathway or complex as the final result.

Generally, the local network alignment strategy is similar to that for local sequence alignment, beginning with a seed that can be aligned with high confidence, which is often based on BLAST scores. A scoring scheme is defined, often based on an explicit evolutionary model, and then the alignment is extended outwards from the seed along network edges, incorporating as many other protein pairs as possible and optimizing on the score. NetAligner^22^, for example, assumes that interacting proteins evolve at similar rates as part of scoring edge mismatches and gaps. MaWISH^23^ formulates an evolutionary model consisting of three events: match, mismatch, and duplication, which are used to develop a scoring scheme for optimization and thresholding. The explicit use of an evolutionary model to generate a scoring scheme is not novel; as with sequence alignment, any network alignment method implies an evolutionary model. However, as protein-protein interaction network evolution remains a largely mysterious process, the evolutionary models underlying the scoring schemes are diverse.

Otherwise, the local network alignment problem is well defined. The objective is to identify small, well-defined interactomic units – such as protein complexes or pathways – that are analogs within the input networks. However, by focusing on local regions, they may miss global aspects of network evolution. Additionally, as certain topological patterns appear frequently in PPINs, such as cliques and hubs, local network alignments can improperly align subnetworks corresponding to these patterns. This is typically prevented using minimum sequence similarity thresholds, explicitly or implicitly, to block the alignment of proteins with dissimilar sequences.

Global network alignment methods attempt to align all or most of the proteins in two or more PPINs. These methods typically build interactome-wide alignments either by seeding an initial alignment and then extending it or by seeking a global optimum according to some scoring mechanism using methods such as the Hungarian^24^ or the PageRank^17^ algorithms. Global alignments likely have much higher false positive rates than local alignments as they align many more protein pairs, even those for which evidence is weak. Still, global alignment methods have produced network alignments with significant levels of functional similarity between aligned proteins.

The IsoRank algorithms – IsoRank^17^ and its successor IsoRankN^25^ - adopt a global approach to the PPIN alignment problem, formulating a set of mathematical equations and solving them concurrently across the entirety of the two networks, in a manner similar to the PageRank algorithm. The intuition behind the IsoRank algorithm is that two vertices should be aligned if their respective neighbours should be aligned, considering similarities of neighbours and BLAST sequence similarity. To solve for all possible vertex pairings, the problem is reframed as an eigenvector, and approximated using the power method. Once convergence is achieved, the vertices are aligned greedily based on their similarity scores. Neither topological similarity nor an evolutionary model for networks are explicitly incorporated in this approach.

GRAAL^16^ and H-GRAAL^24^ focus on the use of graphlet degree signatures^15^ as a purely topology-based measure of vertex similarity. GRAAL and the related MI-GRAAL^26^ use a seed-and-extend approach aligning in expanding radii from the seed vertices in both input networks, aligning the vertices at each radius greedily. H-GRAAL, like IsoRank, formulates the global network alignment problem as a minimum-weight bipartite matching problem and solves this problem using the Hungarian algorithm. C-GRAAL^27^ uses BLAST sequence similarity in a seed-and-extend approach where nodes with high neighbourhood densities are selected as seeds, greedily aligning their neighbourhoods, and then using a common neighbourhood mechanism to align further.

Alternatively, network alignment algorithms can use evolutionary models to score possible alignments in terms of likelihood, as BLAST does with sequence alignments. Unlike the IsoRank and GRAAL algorithms, Graemlin^28^ and Graemlin 2.0^29^ explicitly formulate a model for network evolution, consisting of four distinct evolutionary events for Graemlin and six for Graemlin 2.0. These models are trained on pre-existing protein orthologies from KEGG^30^, and then used to score potential alignments between networks. However, even using a seed-and-extend method that takes an iterative approach to alignment creation, the number of possible events results in an exponential number of possible steps at each iteration, requiring complicated heuristics to manage algorithm complexity. Furthermore, there is no generally accepted model of PPIN evolution and unlike with bases in sequence alignment, there is no clear synonymity between proteins.

Most PPIN alignment methods have attempted to align pairs of related proteins, analogously to pairs of similar amino acids in protein sequence alignment. However, many proteins are part of orthologous and paralogous groups. This has been only recently treated in network alignment, due to the significant complications it creates in both the design of an algorithm and in the subsequent assessment of the algorithm’s effectiveness. Despite this, a few attempts have been made to create alignment methods that produce many-to-many alignments between proteins; these are exclusively extensions of previous one-to-one alignment methods, such as IsoRankN^25^ and Graemlin 2.0^29^ (which extend IsoRank^17^ and Graemlin^28^ respectively). In both of these cases, the later iteration was shown to be more effective, based on functional enrichment of aligned proteins.

## Results

### Comparison with PPIN Alignment Algorithms

To assess the GreedyPlus algorithm, we tested it, along with several algorithms for PPIN alignment, by aligning available worm and yeast SH3 domain IINs^4^,^5^. We first implemented two naïve alignment algorithms to serve as baselines. The first is a greedy algorithm that aligns vertices solely in descending order of similarity score. The second is a seed-and-extend algorithm that initially picks the highest scoring vertex pair as an initial seed for the alignment. It then extends the alignment along the edges of the two networks by iteratively aligning the highest scoring pair of unaligned vertices connected to already aligned vertices. Thus the seed-and-extend algorithm always aligns two edges every time it aligns two vertices. We also used several other published network alignment algorithms – IsoRank, GRAAL, H-GRAAL, C-GRAAL, and Natalie 2.0^31^. For fair comparison, the algorithms were prevented from aligning domain vertices and ligand vertices to each other; this was done either using negative scores for domain-ligand pairs or the algorithms were re-implemented with only this specific additional constraint added.

**Figure 1:**
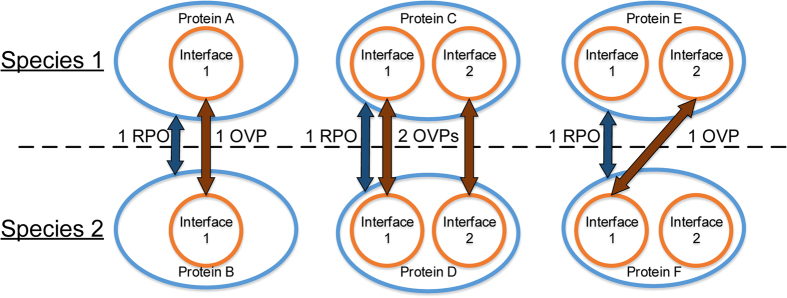
Illustrative examples of represented protein orthologies (RPOs) and orthologous vertex pairs (OVPs). In each of the subfigures, the two proteins are assumed to be orthologous between Species 1 and 2. The orange circles represent specific sites within each protein, depicted as blue ellipses in hypergraph form, and the dark orange arrows represent alignment of the two interfaces. Each pair of aligned interfaces between the two orthologous proteins is 1 OVP. However, regardless of the number of aligned interfaces between the two proteins, there can only be a maximum of 1 RPO, depicted as a dark blue arrow, indicating that the orthologous relationship between the proteins is represented in the alignment.

We compare these algorithms’ performance based on three metrics. The first two – represented protein orthologies (RPO) and orthologous vertex pairs (OVP) are measures of how well the algorithms reproduce known orthologous relationships (see Fig. 1). An RPO is a pair of orthologous proteins, one from each species aligned, which depends on alignment of at least one pair of corresponding interfaces (vertices). An OVP is a pair of aligned interfaces that implies a pair of orthologous proteins; thus, #RPO ≤ #OVP by definition for any alignment. Finally, we ask how well the networks align topologically, by counting the number of edges aligned (EA).

As IsoRank and Natalie 2.0 use BLAST protein similarity as their only similarity feature, our first comparison uses only BLAST protein similarity. C-GRAAL uses BLAST score among others, so we include it in these tests. The Edge Alignment Weight parameter for GreedyPlus was set to 0.5 after testing with several values (see Discussion). The Greedy, GreedyPlus, and IsoRank algorithms all align similar numbers of orthologous vertices (20, 18, and 19 OVPs respectively, out of a maximum of 22, see Table 1), capturing most of the orthologous protein pairs (RPO) present in our worm and yeast datasets (13, 14, and 12 respectively, out of a maximum of 16).

**Figure 2:**
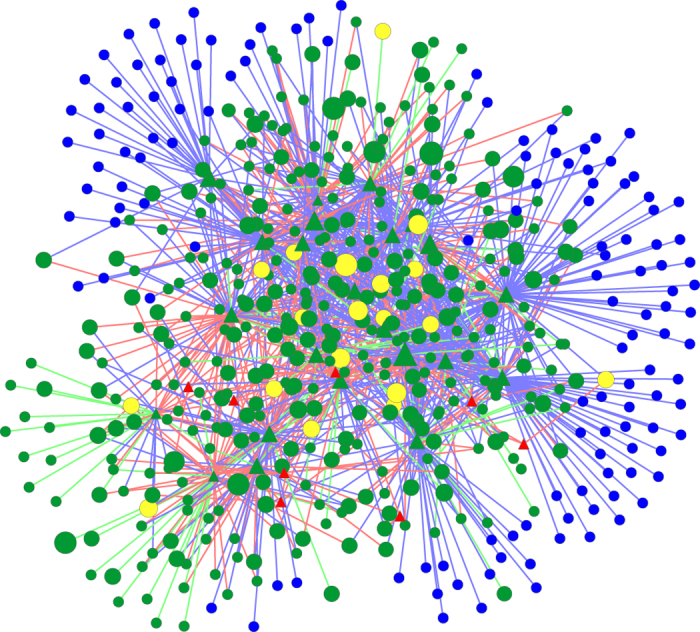
IsoRank alignment of worm and yeast SH3-mediated IINs, using only protein BLAST. Domain interfaces are represented by triangular vertices, ligand interfaces by circular vertices. Yellow vertices are aligned and from orthologous proteins (OVPs), green vertices and edges are aligned but not orthologous, red are unaligned from worm, blue are unaligned from yeast. Vertex size indicates score. The fact that IsoRank largely ignores edge alignment is reflected in the low number of green edges. While there are more blue edges and nodes than red, due to the larger size of the yeast network, there are no large clusters of green (aligned regions).

While the greedy algorithm was successful at aligning vertices from orthologous proteins, the low (27 out of a maximum possible 466, 6%) number of edges aligned implies that it is a poor network alignment strategy. This may be expected, as the algorithm does not consider edges. The IsoRank algorithm also aligns edges poorly (96 of 466 EAs, 21%), as it primarily focuses on the alignment of similar nodes. The bipartite nature of the networks also causes unusual behaviour: the R similarity score in IsoRank fails to properly distribute itself throughout the networks, instead oscillating between domains and ligand sites rather than converging to a stable state. An examination of the resulting IsoRank alignment (see Fig. 2) reveals no connected concentrations of aligned vertices and edges, and thus no regions of similar topology between the *C. elegans* and *S. cerevisiae* networks.

Similar to IsoRank, the C-GRAAL algorithm also struggles with the topology of the network. The seeds it finds are invariably ligand sites adjacent to multiple domains, as domains have much higher degrees. However, domains share few common neighbours due to their binding specificity, and ligand sites have generally few neighbours. This limits the alignment expansion, based on its core common-neighbours concept, to less than half of the final alignment.

The seed & extend and Natalie 2.0 algorithms captured very few orthologies (1 and 0 of 16 RPOs, respectively) as they are primarily focused on edge alignment. Seed & Extend makes an early unrecoverable error, beginning alignment at the periphery of the worm network and then rapidly dead-ending, aligning only a total of ten vertices (see Supplementary Figure 1 and Supplementary Figure 2). Natalie 2.0 utilizes a scoring scheme focused on maximizing edge-correctness, on which it performs well at the expense of orthology recovery, indicating that simply maximizing network overlap is insufficient for reproducing known biological relationships.

**Figure 3:**
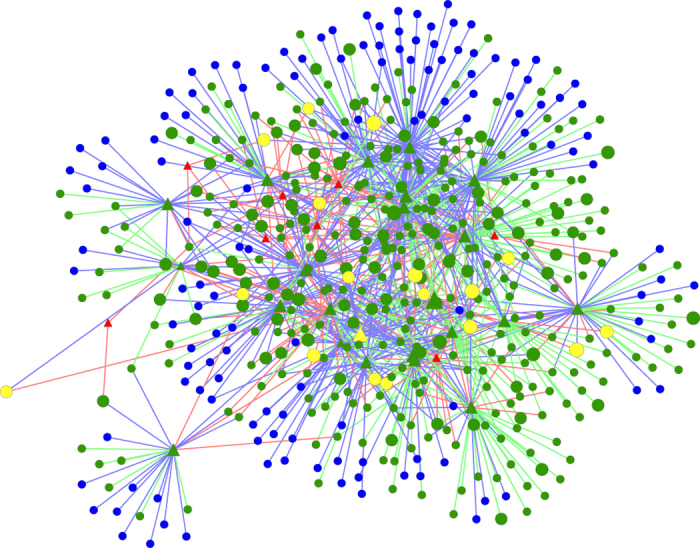
GreedyPlus alignment of worm and yeast SH3-mediated IINs, using only protein BLAST, EAW = 0.5. Domain interfaces are represented by triangular vertices, ligand interfaces by circular vertices. Yellow vertices are aligned and from orthologous proteins (OVPs), green vertices and edges are aligned but not orthologous, red are unaligned from worm, blue are unaligned from yeast. Vertex size indicates score. The GreedyPlus algorithm aligns many more edges than IsoRank, resulting in many fewer blue and red edges, as they are replaced by half as many green edges. However, there are still no large clusters of green, with red and blue edges dispersed throughout the alignment, indicating that interaction rewiring is both common and distributed.

Finally, GreedyPlus performs best on RPO and second-best on both OVP and EA (14 of 16, 18 of 22, and 291 of 466 respectively). It is also the only algorithm that performs evenly across the three metrics, with performance each at >60% of max, and thus generally performs the best in this comparison (see Fig. 3).

**Figure 4:**
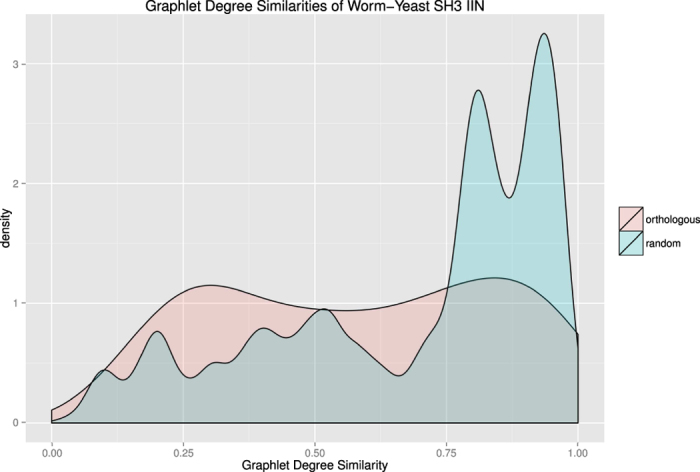
A density plot of graphlet similarity scores between orthologous vertices (in pink) and random vertex pairs (in blue). Orthologous vertex pairs do not demonstrate a characteristic graphlet similarity score; as such, graphlet similarity has reduced power in correctly aligning vertices. Note that many random vertex pairs have high graphlet similarity in the IINs under study; this is due to the prevalence of leaf vertices, which tend to exhibit similar graphlet degree vectors.

The GRAAL and H-GRAAL algorithms rely on a single vertex similarity feature, known as the graphlet degree signature^16^. Thus our second comparison uses only graphlet degree vertex similarity across all compared algorithms (Table 2), including C-GRAAL as it was also tested with just graphlet degree signature. This vertex similarity measure results in poor vertex alignment performance across all algorithms. For example, the GRAAL algorithm identifies no orthologous vertex pairs, though it does have a similar execution time as GreedyPlus. The generation of the graphlet degree signature for a given vertex involves counting the number of 2-, 3-, 4-, and 5-vertex graphlets in which the vertex participates. However, of the 29 graphlets of such size, 20 of them contain odd cycles not present in bipartite networks. This reduces the number of graphlet orbits, and the length of the graphlet degree signature vector, from 72 to 20. Due to this loss of resolution, the GRAAL algorithm loses power in discriminating between vertex pairings (see Fig. 4). Furthermore, the exaggerated spoke-hub network of IINs in comparison to PPINs, for which the GRAAL algorithm was designed, results in the GRAAL algorithm preferring to align non-orthologous vertices to orthologous ones.

H-GRAAL, which focuses on aligning vertices, also fails due to the loss of resolution, while the C-GRAAL algorithm suffers from the same issues as in the earlier tests. Thus, for graphlet degree, different network types require different topological considerations to be aligned properly.

### Incorporating More Similarity Features

As seen in our comparisons above, the choice of similarity feature can dramatically affect algorithmic performance across a range of algorithms for a given network. In particular, the simple Greedy algorithm, using a highly informative similarity feature (BLAST), was more successful at recovering orthologous protein relationships than the more advanced GRAAL algorithm using a poor similarity feature (graphlet degree) for our IIN (13 RPOs, 20 OVPs vs. 0 RPOs, 0 OVPs, respectively). To investigate the information content of diverse similarity features and their impact on network alignment, we gathered 29 vertex similarity features, based on sequence, functional annotation, and topological characteristics (see Supplementary Table 1, Methods), and attempted alignment with GreedyPlus using all these features, equally weighted with each other and the Edge Alignment Weight (EAW, see Methods).

In this comparison, with 29 equally weighted similarity measures, the H-GRAAL algorithm performs best in aligning orthologous vertices, with 11 RPOs and 15 OVPs, but it aligns only 10% (47) of the edges (Table 3). This performance is very similar to that of the Greedy algorithm in all respects, suggesting that edge alignment in H-GRAAL is largely by chance. Other than Natalie 2.0, which produces the exact same alignment as in the first comparison (Table 1), the Seed & Extend algorithm aligns the fewest orthologous vertices (2 OVPs, 9%,), but aligns the most edges (306 EAs, 65%). As every pair of vertices the algorithm aligns must be connected to two previously aligned vertices, the algorithm tends to generate a high number of edge alignments, but this inflexibility causes it to ignore possible vertex alignments supported by high similarity scores when no neighbours have yet been aligned. The importance of the input similarity feature is shown by the improved performance of GRAAL due to the introduction of more informative similarity features, and the decreased performance of IsoRank, due to the dilution of the highly informative BLAST similarity feature. However, GreedyPlus had the best balanced performance in both properly aligning vertex pairs (44% RPOs, 45% OVPs) and the number of aligned edges (51% EAs).

### Parameter Weight Tuning

Having established GreedyPlus’ performance using naïve parameterizations on the weights of each of the 29 similarity measures, we investigated how improved parameterizations would affect alignment quality. We used a random-restart hill-climbing strategy to search the high-dimensional parameter space for local maxima in orthology recovery (see Methods). This strategy was applied to all 29 similarity features plus the edge alignment weight together (resulting weights listed in Supplementary Table 1). Using this procedure, we found a set of parameters that can recover all possible orthologies (16 RPO, 21 OVP) with a high number of edges aligned (210/466, 45%).

However, several local maxima existed that each resulted in similarly high orthology recovery. Also, some parameters are similar to each other, thus not all 29 may be required. To address the possibility of overfitting, we gradually reduced the number of parameters while repeating the search/optimization procedure. In so doing, we found a set of 6 parameters still capable of producing high-quality alignments (16 RPO, 22 OVP, 218/466 or 47% EA), as shown in Table 4.

Sequence similarity features account for ~64% of the overall parameter weighting. Topological considerations, including the closeness similarity features and the edge alignment weight – which is not a similarity feature and can be applied multiple times to the same pair of potentially aligned vertices – account for ~30%.

Including closeness and the edge alignment weight in addition to the sequence similarity features improved orthology recovery. When closeness was removed, the resulting alignment produces only 13 RPO, 17 OVP, and 231 EA. Similarly, setting the edge alignment weight to zero results in a poorer alignment, in particular with edges: 13 RPO, 19 OVP, 34 EA. The small weight assigned to the functional similarity feature Topological Clustering Semantic Similarity (TCSS)^32^, however, is insignificant; setting it to zero did not change the overall alignment performance, despite TCSS being weighted relatively highly when optimization was performed using all assembled features (Supplementary Table 1). Removing all non-sequence similarity features (i.e. using only BLAST and Smith-Waterman) results in 13 RPO, 19 OVP, 29 EA. Given the decreased performance using just sequence similarity features, we conclude that non-sequence similarity features are useful in determining the similarity between vertices for the purposes of network alignment.

### A Zoom-in

**Figure 5:**
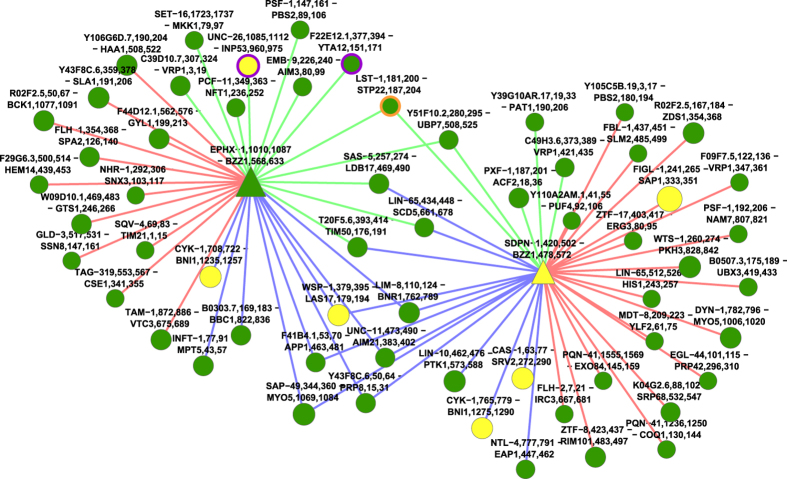
A zoom-in of the “optimal” GreedyPlus alignment of worm and yeast SH3-mediated IINs, consisting of the two yeast BZZ1 vertices and all their neighbours. Domain interfaces are represented by triangular vertices, ligand interfaces by circular vertices. Yellow vertices are aligned and orthologous; green vertices and edges are aligned, red are unaligned from worm, blue are unaligned from yeast. Vertex size indicates score. The two EAW contributors to EPHX-1,1010,1087 - BZZ1,568,633 are outlined in purple; the EAW contributor to SDPN-1,420,502 - BZZ1,478,572 is outlined in orange.

As a snapshot of how GreedyPlus works in practice, we zoom in on the yeast protein BZZ1 (see Fig. 5), which has two domain vertices in our dataset. BZZ1 is a recruiter protein involved in regulating actin polymerization^33^, and is an ortholog of the worm protein SDPN-1; Gene Ontology identifies both genes as involved in endocytosis^34^. In this alignment, performed by GreedyPlus using its tuned similarity weights, one of the BZZ1 domains is aligned to SDPN-1’s single SH3 domain. However, because GreedyPlus cannot perform one-to-many alignments, BZZ1’s other SH3 domain is aligned to EPHX-1, which is not an identified ortholog. Neither SDPN-1 nor EPHX-1 are among BZZ1’s top BLAST scores, ranking 9^th^ and 11^th^ among our dataset; however, the other similarity features and the Edge Alignment Weight drive up their priority in alignment.

Interestingly, the EPHX-1 – BZZ1 alignment was performed first, as the neighbouring aligned pairs F22E12.1,377,394 - YTA12,151,171 and UNC-26,1085,1112 - INP53,960,975 boost its score, via the EAW, by ~36%, illustrating the additive effect of the EAW. Subsequently, the vertices LST-1,181,200 and STP22,187,204 are aligned, partially on the strength of the EAW from EPHX-1 – BZZ1, which then promotes the alignment of SDPN-1 to BZZ1. A number of other orthologous node alignments occur in the immediate neighbourhood, but do not contribute any EAW to the BZZ1 domain alignments because they are not adjacent in either the worm or yeast networks. For example, WSP-1 and LAS17 are orthologs, but while LAS-17 interacts with BZZ1 in yeast, its worm ortholog WSP-1 does not interact with either SDPN-1 or EPHX-1 in our SH3 dataset, nor is such an interaction found in the interaction data iRefIndex^35^, hinting at a previously undetected interaction.

**Figure 6:**
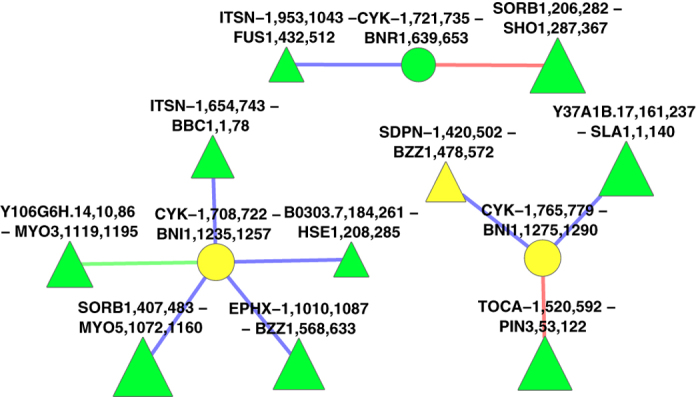
A zoom-in of the “optimal” GreedyPlus alignment of worm and yeast SH3-mediated IINs, consisting of the three worm CYK1 vertices and all their neighbours. Domain interfaces are represented by triangular vertices, ligand interfaces by circular vertices. Yellow vertices are aligned and orthologous; green vertices and edges are aligned, red are unaligned from worm, blue are unaligned from yeast. Vertex size indicates score.

We also observe that while BNI1 is an interaction partner with BZZ1, with two sites targeted by the two BZZ1 SH3 domains, its worm ortholog CYK-1 does not interact with either EPHX-1 or SDPN-1. This non-interaction is also supported by iRefIndex. In our worm network, the respective CYK-1 sites are targeted only by Y106G6H.14 and TOCA-1, neither of which have functional annotations in GO, though TOCA-1 is indicated to be involved in endocytosis as well^36^,^37^ (see Fig. 6). This extensive interaction rewiring suggests that IIN alignment approaches based on maximizing topological overlap may not be appropriate in identifying orthologs.

### Yeast Subspecies Alignments

In addition to the *C. elegans* to *S. cerevisiae* IIN alignment, we tested GreedyPlus on published predicted SH3 IINs from 18 different yeast species^38^ (see Methods). All 18 networks were pairwise aligned, using both the full set of features and the reduced set identified above. TCSS was removed as a similarity feature to remove circularity, as most GO annotations for these yeast species’ proteins are predicted via orthology with *S. cerevisiae*. The feature weighting identified in the above described optimization for GreedyPlus on *C. elegans* and *S. cerevisiae* was used.

**Figure 7:**
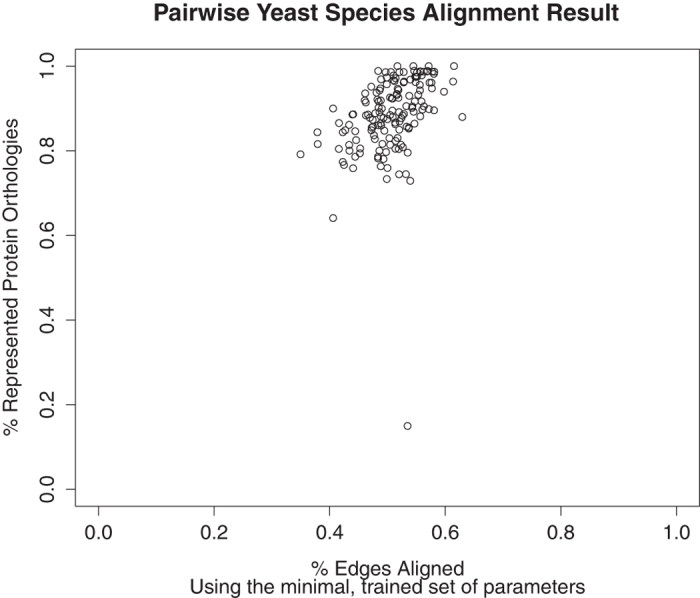
Percent RPO and EA achieved for pairwise yeast species alignments. Using the optimized parameters from Table 4, GreedyPlus was run on each pair of yeast networks (see Methods). The percent of represented protein orthologies and edges aligned for each alignment was retrieved and plotted on the same scale.

**Figure 8:**
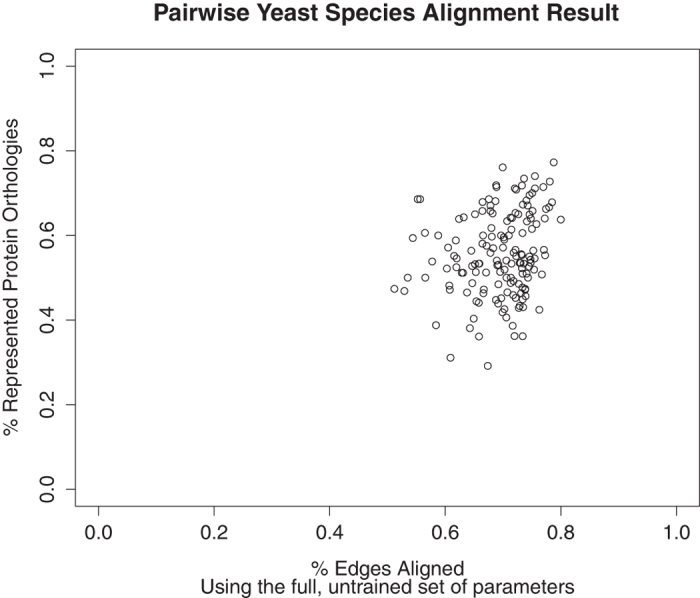
Percent RPO and EA achieved for pairwise yeast species alignments. Using the full set of similarity features and no optimization, GreedyPlus was run on each pair of yeast networks (see Methods). The percent of represented protein orthologies and edges aligned for each alignment was retrieved and plotted on the same scale.

As these species are more closely related than *C. elegans* and *S. cerevisiae*, we found, as expected, that GreedyPlus is able to recover more orthologous pairs in these pairwise alignments. When using a minimal set of similarity features with optimized weights (see Table 4), GreedyPlus alignments almost always recovered more than 70% of the known orthologous protein pairs while still maintaining a high percentage of edges aligned (mean 50.6% of maximum possible, see Fig. 7). Using all the gathered similarity features, except TCSS (26 features), GreedyPlus still performed well, aligning an average of 56% of orthologous protein pairs (see Fig. 8).

In both cases, a high percentage of the edges were aligned; notably, more edges were aligned when the full set of 26 similarity features were considered. This result is contrary to what would be expected; given equal weights, with more similarity features, the relative weight of the EAW is decreased from 1/5^th^ of the overall scoring function to 1/26^th^. This suggests that the additional similarity features – almost all based on network topology – increase the alignment of edges by promoting the alignment of topologically similar vertices. This may be due to the inferred nature of these networks from relatively closely related species which lead to more topologically similar networks.

## Discussion

We have described GreedyPlus, a network alignment algorithm that is effective, flexible in terms of input data, and fast, outperforming traditional network alignment methods in aligning IINs. With feature optimization, made easier by GreedyPlus’ speed, we identified a set of data features and their weights that proved highly effective in guiding network alignment.

Unlike other network alignment algorithms, GreedyPlus explicitly specifies a trade-off between a vertex alignment and edge alignment via the EAW parameter. This means that *a priori* knowledge about the networks being aligned can be used to control alignment. Lower EAW values should be more suitable for dissimilar networks, to force the algorithm to focus on vertices, whose similarity scores should already be sufficiently differentiated to distinguish proper from improper alignments. On the other hand, higher EAW values should be more suitable for highly similar networks, resulting in a stricter alignment, which would highlight the few areas of difference.

This feature makes the GreedyPlus algorithm suited for evaluating the relative importance of vertex versus edge alignment in network alignments. Identifying the correct parameterisation for the alignment of different types of networks is in itself an interesting research problem capable of informing us on how networks evolve.

Another important feature of GreedyPlus is that it is mostly agnostic to the topological nature of the networks being aligned, other than the assumption that neighbours of aligned vertices should more likely be aligned themselves. As our SH3 domain data set does not contain domain-domain or ligand-ligand interactions, the IINs we studied were bipartite, which confounded several of the algorithms tested. Though the current GreedyPlus implementation is specialized to handle bipartite networks, it is not dependent on the bipartition, and the approach could be adapted to different network types. For example, domain-domain interactions are possible with other domains, such as SAM and coiled-coil, and so IINs are not necessarily bipartite.

**Figure 9:**
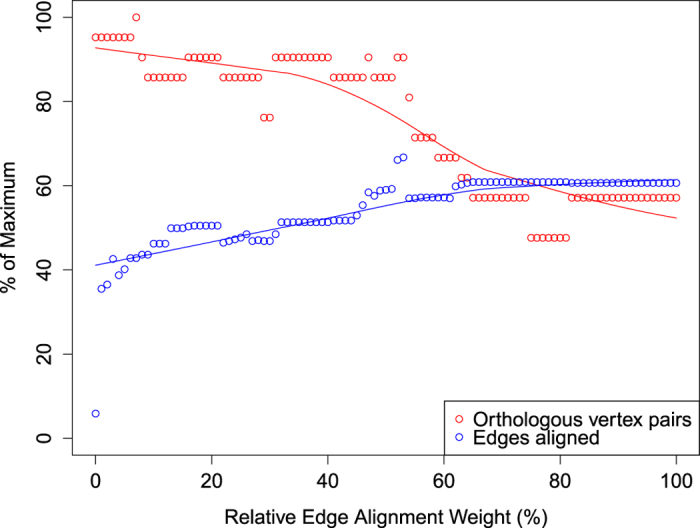
Trade-off in GreedyPlus performance between orthologous vertices aligned and edges aligned. Using BLAST score between proteins as the only similarity feature, we ran GreedyPlus with the edge alignment weight set at values ranging from 0% to 100% of the protein BLAST score weight, and plotted its performance. If the plots were tightly correlated, it would indicate that successfully aligning networks topologically would be equivalent to aligning vertex pairs successfully. However, we observe a distinct trade-off between aligning edges and aligning orthologous vertices.

Though we lack sufficient IIN data to make a general statement, we observed a trade-off between the alignment of biologically verified vertex pairs and the alignment of edges with a number of algorithms, including our own. Notably, an increase in the number of edges aligned did not necessarily lead to an increase in the number of vertices properly aligned. Some brief experiments with the Edge Alignment Weight parameter showed that, with GreedyPlus, attempting to maximize the number of aligned edges results in a distinct decrease in the number of properly aligned vertices (see Fig. 9).

Despite this trade-off, our results show that including topological similarity features improves the orthology predictions of network alignment, demonstrating their relevance, and hinting that network alignment may complement sequence alignment as a bioinformatics tool to study evolution, but the significance of edge alignment in constructing network alignments is unclear. While aligning two edges implies similarity between their endpoints, simply maximizing the number of edges aligned clearly does not result in a biologically relevant and informative alignment. Though it would be a simple extension, GreedyPlus does not currently implement edge similarity features, and treats all interactions as functionally identical, because while some are available, such as PPI confidence estimates^39^, binding affinity, tissue specificity, or the types of interacting residues, they are not currently prolific enough to be generally useful. It is possible that with discriminatory information about the interactions that edges represent, a method based on optimizing edge alignments may prove effective for aligning entire networks as well. Using this information, a PPIN alignment algorithm, for example, could preferentially align two SH3-mediated interactions, rather than an SH3-mediated interaction and a WW-mediated interaction, or could avoid aligning a structural interaction to a transient enzymatic interaction.

We selected a broad set of similarity features to investigate their utility in network alignment. Sequence similarity, in particular BLAST-based, is ubiquitous in network alignment research; ligand sequences, however, are too short for BLAST, so Smith-Waterman was used instead. GO functional annotations are often used to validate network alignments; we were interested in examining whether they could be used as an input feature as well. Topological features other than graphlet signatures have had some treatment in the literature; Kuchaiev *et al.*^26^ have previously evaluated graphlet signature, vertex degree, clustering coefficient, and eccentricity. As such, we opted to incorporate many different topological features to assess their utility (see Supplementary Table 1). In general, the features are of two types: centrality or clustering. Average shortest path length, betweenness, closeness, eccentricity, radiality, and stress are all measures whether a vertex is located in the centre or on the periphery of a network. The alignment of a central vertex to a peripheral vertex would, given our current knowledge of network evolution, imply a highly improbable evolutionary history, involving a large redistribution of vertex and edges about a previously peripheral vertex. Degree, graphlet signature, neighbourhood connectivity, stress, and topological coefficient are all measures of connectedness in the neighbourhood around a vertex. These measures should distinguish a vertex in a highly connected neighbourhood from one in a sparse neighbourhood, providing regional information to guide alignment. Clustering coefficient was not used, as the vertices in a bipartite graph always have a clustering coefficient of zero^40^.

Notably, using BLAST score for full-length proteins alone as the single distinguishing similarity feature results in substantial orthology recovery in the alignment (see Table 5). Conversely, using the sequence similarity scores of the vertices - BLAST for domains and Smith-Waterman for ligands – alone resulted in no orthology recovery. Various combinations of the topological and functional similarity features, as determined using TCSS^32^, also resulted in low orthology reproduction. This seems to suggest that while non-sequence-based information has a role to play in network alignment, its direct contribution is not obvious.

Many measures of network alignment quality are also dependent on BLAST similarity. In addition to orthologs, some measure of coherence between the GO terms of aligned vertices is often used to verify the biological quality of an alignment. However, many GO terms are inferred from another protein based on sequence similarity, either directly or indirectly. Even experimentally derived GO terms may be subject to BLAST-derived confirmation bias, as experimental design could be guided by BLAST results. Notably, while using functional features alone generates an alignment with orthology reproduction (see Table 5), they played close to no role in our optimized parameter set (see Table 4); this may be explained by a duplication of information between sequence- and function-based similarity features. If network alignment is to serve as an independent tool alongside BLAST, the development of assessment measures that don’t involve BLAST-based confirmation bias will be essential.

Finally, network alignments, consisting of at least two networks plus the alignment between them, present a challenging visualization problem. Even with the relatively small, sparse IINs, alignments produce networks exhibiting the same “hairball” nature characteristic of the PPIN visualization problem^41^. For instance, it would be useful to evaluate compound graph and hypergraph visualizations, similar to what is presented in Fig. 1. With the proliferation of network alignment research, improved visualization tools will be critical for the interpretation of generated alignments.

## Conclusions

GreedyPlus is a novel algorithm useful for the alignment of interface-interaction networks, compatible with a range of vertex similarity measures. While vertex sequence information is dominant in its ability to align vertices properly, topological information is useful for improving alignment performance, even if it is of low utility in isolation. We identify a reduced set of information types and a weighting of these types that can be used to generate relatively high performance alignments. The algorithm and our evaluation framework will be used to further investigate network evolution and how to best align biological networks.

## Methods

### Algorithm

We created a fast algorithm for the alignment of IINs, GreedyPlus, using a local network alignment approach. Additionally, alignments generated by GreedyPlus can easily be compared, on a stepwise basis, to identify when and why each alignment tuple was formed (or not), allowing us to specifically query how changes in parameterization and input data may lead to differences in the resulting alignment. Further, a key research question for network alignment is how to balance vertex-specific information versus topological information. The critical characteristic of network alignment is that edges must be aligned in addition to vertices; an alignment that has no aligned edges is, fundamentally, not a network alignment^42^. However, while there is a plethora of biological information regarding proteins, there is a dearth of information on protein-protein interactions that would assist in guiding or verifying an alignment. Thus, to investigate the interrelation and relative importance of vertex versus edge alignment, GreedyPlus explicitly models a balance between these two elements.

An interface interaction network can be modelled as an undirected graph *G*, consisting of a vertex set V and an edge set E, where each edge is a tuple of two vertices (*v*_*1*_, *v*_*2*_). An alignment of two PPINs *G*_*1*_ and *G*_*2*_ is thus a set of 2-tuples *A *= {[*u*_*1*_, *v*_*1*_], [*u*_*2*_, *v*_*2*_], …, [*u*_*i*_, *v*_*i*_]}, where *u*_*i*_ ϵ *V*_*1*_ ϵ *G*_*1*_ and *v*_*i*_ ϵ *V*_*2*_ ϵ *G*_*2*_, if the alignment is one-to-one. In this context, the goal of GreedyPlus, like most other biological network alignment algorithms, is primarily to align vertices [*u*_*i*_, *v*_*i*_] such that biological inferences can be drawn about one vertex from the other based on their alignment. If these vertices represent interfaces, such an inference might be that they are orthologous, or that they mediate functionally similar interactions, or that they evolved to occupy similar positions in their respective networks under similar selective pressures.

Though generalizable to other networks, GreedyPlus’ current implementation is tailored specifically to accommodate bipartite peptide-recognition module-mediated IINs, reflecting the current availability of IIN data. These networks, being bipartite, can be modelled in a similar manner as general IINs, as an undirected graph *G*, with a vertex set *V* = {*D*, *L*}, where *D* and *L* are the sets of vertices representing peptide recognition domains (e.g. SH3) and ligands respectively, and ∀ *v* ϵ *G*, *v* ϵ *D* or *v* ϵ *L*. An alignment *A* of such networks is then restricted such that for each tuple [*u*, *v*] ϵ *A*, either (*u* ϵ *D* Λ *v* ϵ *D*) or (*u* ϵ *L* Λ *v* ϵ *L*).

The key intuition behind the GreedyPlus algorithm is that the presence of interaction is itself a biological evidence source pointing towards an orthologous relationship between a pair of proteins. That is, if there exists (*u*_*1*_, *u*_*2*_) ϵ *G*_*1*_ and (*v*_*1*_, *v*_*2*_) ϵ *G*_*2*_, and it is known that u_2_ and v_2_ are orthologous, then we can infer that u_1_ and v_1_ are more likely to also be orthologous. Furthermore, if there also exist (*u*_*1*_, *u*_*3*_) ϵ G_1_ and (*v*_*1*_, *v*_*3*_) ϵ *G*_*2*_, this would provide even stronger evidence; thus the more edges that would be aligned by aligning vertices u_1_ and v_1_, the more likely that this is a good alignment of vertices.

**Figure 10:**
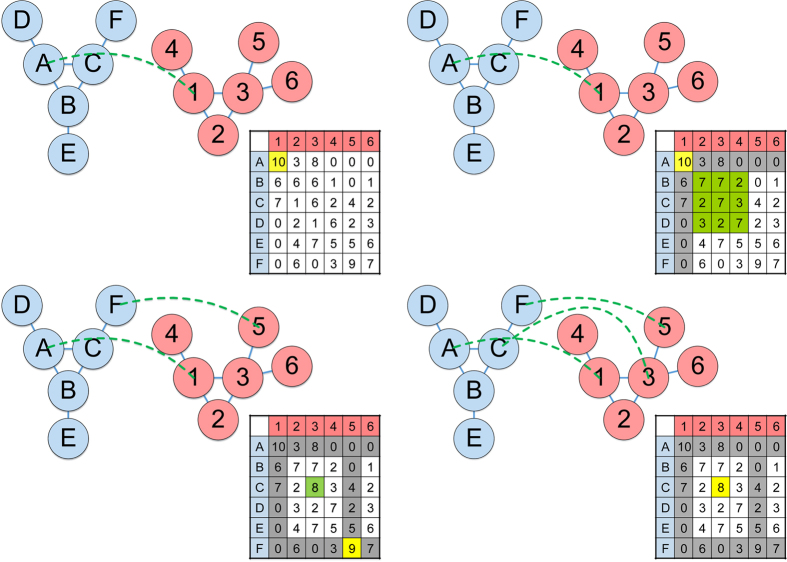
A simple example of GreedyPlus in action. First, GreedyPlus finds the highest scoring pair of vertices (in yellow), in this case the pair (*A*, *1)*, and aligns them. Then the similarity matrix is updated, with the scores of all pairs of all neighbours of just-aligned vertices (in green) incremented by the Edge Alignment Weight (in this case, 1). Using the updated similarity matrix, GreedyPlus iterates until all vertices are aligned. In this example, the third vertex alignment [*C*, *3*] is made as a result of the Edge Alignment Weight increasing its similarity score; otherwise, the pairing [*E*, *3*] would have been made instead.

**Figure 11:**
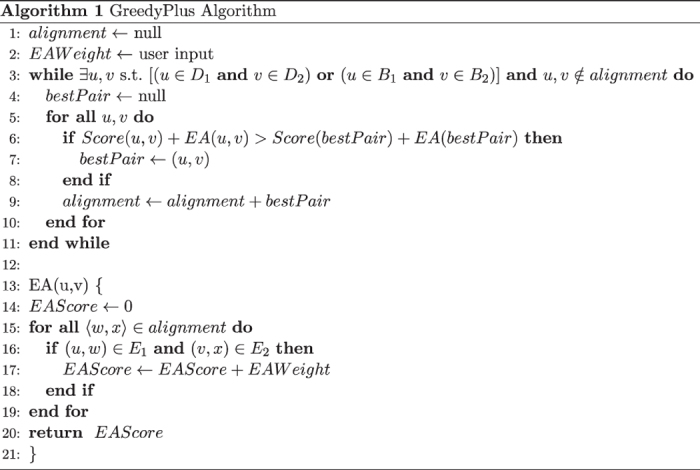
Pseudocode for the GreedyPlus algorithm. This pseudocode is not optimized, for clarity purposes. Note that EAW has an additive effect; the more edge alignments that support a given vertex alignment, the higher the corresponding score is boosted.

The GreedyPlus algorithm is essentially a greedy algorithm that iteratively aligns pairs of vertices in descending order of similarity, defined by a given similarity score. However, when aligning two vertices, GreedyPlus also considers the number of edges that would be aligned if the vertices were aligned, strengthening the respective vertex pair similarity score with more edges aligned (see Fig. 10 and Fig. 11). Thus, GreedyPlus will prefer to align vertex pairs that also align edge pairs over those that do not if the difference in similarity is small, but will align highly similar vertices irrespective of network topology. The preference of the algorithm in aligning edges or maximizing vertex similarity can be controlled using a defined parameter, named the *edge alignment weight (EAW)*, providing flexibility and enabling investigation of the relative importance of aligning vertices versus edges.

The edge alignment weight (EAW) determines how strongly GreedyPlus prioritizes the alignment of edges compared to vertices. When the EAW is set to zero, GreedyPlus behaves identically to the greedy alignment algorithm, as it will ignore the alignment of edges. When the EAW is set to ∞, GreedyPlus behaves similarly to a seed-and-extend algorithm (see below), always choosing to align two edges whenever possible as the EAW will overwhelm any preference in aligning vertices, with the exception that it can resume alignment even if edge extension possibilities are exhausted. By tuning the EAW parameter to intermediate values, the preference between vertex or edge alignment can be balanced.

A naïve implementation of GreedyPlus runs in worst-case O(|*D*|^3^|*E*| + |*L*|^3^|*E*|) time, though given the nature of domains and ligands, |*L*| >> |*D*|. In practice, GreedyPlus takes approximately two seconds to align two networks with |*L*| = 500 and |*E*| = 600, implemented in Java on a 3.4 GHz processor.

### Network Creation

The *S. cerevisiae* and *C. elegans* networks were created using interaction data from Tonikian *et al.* and Xin *et al.*^4^,^5^. 24 SH3 domains were identified in *S. cerevisiae*, their 853 interactions were experimentally identified, and then 497 ligand targets for those interactions were predicted. Similarly, 33 SH3 domains, 466 SH3-mediated interactions, and 433 SH3 ligands were identified in *C. elegans*. Each SH3 domain was represented by an individual vertex, with the exception of the first two SH3 domains on the Sla1 protein, which were treated as a single domain in the original prediction procedure, as the two domains could not be purified separately. These networks cover approximately half of the SH3 domains in each species; the remainder failed for various experimental reasons and were thus excluded from our work.

Each predicted interaction included a target peptide sequence of length 15. When these peptide targets occupied non-overlapping positions in the target proteins, each peptide target was as an independent binding site represented as an independent vertex. When binding sites overlapped, they were merged, when possible, into a singular vertex representing no more than 30 amino acids. Overlapping binding sites with a combined length of more than 30 amino acids were manually separated into multiple vertices with minimum sequence overlap between vertices.

The interaction networks for the other yeast species were similarly created using interaction data from Sun *et al.*^38^. Each network contains approximately 500 predicted interactions, generated by using the 30 position weight matrices created for 24 *S. cerevesiae* SH3 domains and mapping them to each yeast proteome in which an orthologous SH3 domain exists. The networks for three species – *S. paradoxus*, *S. mikatae*, and *S. bayanus* – the only three datasets sourced from Fungal Comparative Genomics (original source: Kellis *et al.*^43^), were excluded due to unusual performance. In particular, pairwise alignments between the remaining 20 yeast species had an average protein orthology recovery rate of 56% and a minimum of 29%, compared to just 24% for alignments between the three excluded species and the remaining 20 yeast species.

### Orthology Data

The orthology dataset for *S. cerevisiae* and *C. elegans* was created from a union of orthology mappings retrieved from Ensembl^44^, Inparanoid^45^, and OrthoMCL^46^. The orthology datasets for the yeast species were produced by Wapinski *et al.*^47^.

### Similarity Feature Data

We began with 29 similarity features for assessing every pair of vertices from two separate networks. Sequence similarity was calculated between every pair of proteins using BLAST-P, taking both the raw score and the coverage as features, as well as every pair of domains. Sequence similarity between ligand sites was calculated using the Smith-Waterman algorithm with the BLOSUM62 scoring matrix, as implemented by JAligner^48^.

Functional similarity was calculated between proteins using TCSS^32^, taking the biological processes, cellular components, and molecular function scores as three separate similarity features. Graphlet degree similarity was calculated between all SH3 domain and between all ligand site vertices separately, as described by Przulj *et al.*^49^, as were the remaining 18 similarity features – betweenness, closeness, degree, eccentricity, neighbourhood connectivity, radiality, stress centrality, and topological coefficient. Raw values for these features were obtained using the NetworkAnalyzer plug-in in Cytoscape^50^,^51^, and a raw similarity value was calculated for each pair of domains [*i*, *j*], *i* ϵ *D*_*1*_, *j* ϵ *D*_*2*_, *raw*_*i,j*_ = max(*score*_*x*_ – *score*_*y*_) – (*score*_*i*_ – *score*_*j*_) ∀ *x* ϵ *D*_*1*_, ∀ *y* ϵ *D*_*2*_, and then normalized logarithmically to the interval [0,1] using the formula: ∀ *x* ϵ *D*_*1*_, ∀ *y* ϵ *D*_*2*_, *adj*_*i,j*_ = log(*raw*_*i,j*_)/log(max(*raw*_*x,y*_)). Similarity scores between ligand vertices were calculated similarly.

### Parameter Training Procedure

The parameter training procedure used was a random hill-climbing heuristic, designed to find parameter sets that maximized the orthologies found (RPOs). For each set of similarity features trained, we randomly generated a weight parameter in the interval [0,1], and generated a corresponding alignment. We then randomly incremented or decremented the first parameter by a step value if the new value would remain within the interval [0,1] and generated a new alignment. If the first alignment had more RPOs, then the parameter change was reversed and another parameter chosen to be incremented or decremented. Otherwise, the new parameterization was kept, and the parameter stepping repeated until no further improvement could be achieved. Then every other parameter would be retested for possible improvement via incrementation or decrementation.

This process was continued until no parameter could be either incremented or decremented to improve the orthology reproduction of the alignment produced. This procedure was iterated four times, using increasingly precise step sizes: ^4^√0.01 ≈ 0.32, 0.1, 0.03, and 0.01, until convergence was achieved, resulting in a parameterisation at a presumed local maximum for orthology recovery.

For each set of similarity features used, the training procedure was iterated at least 5,000 times, producing at least 5,000 locally optimal parameter sets.

### Similarity Feature Reduction

To reduce the full set of similarity features to a smaller set, redundant similarity features were identified by calculating Euclidean distances between each similarity feature matrix and performing principal component analysis. Similarity features that were highly similar to another feature (Euclidean distance with another similarity feature < = 0.10) were then removed, with preference given to removing the feature most similar to the other remaining features. The following features were removed: BLAST coverage, TCSS cellular component, and TCSS biological process for proteins, betweenness, BLAST coverage, eccentricity, and radiality for domains, average shortest length path, betweenness, eccentricity, degree, radiality, and stress for binding site.

To further reduce the similarity feature set, GreedyPlus was re-optimized with the remaining 18 similarity features to identify features that could be removed without negatively impacting algorithmic performance. The similarity feature given the lowest weight in parameter sets associated with the top 50 results from the training procedure was identified as the most uninformative feature and removed. This process was repeated until an effective minimal set of similarity features was identified, whereby the removal of any additional feature resulted in loss of orthology recovery.

## Additional Information

**How to cite this article**: Law, B. and Bader, G. D. GreedyPlus: An Algorithm for the Alignment of Interface Interaction Networks. *Sci. Rep.* **5**, 12074; doi: 10.1038/srep12074 (2015).

## Supplementary Material

Supplementary Information

## Figures and Tables

**Table 1:** Comparison between GreedyPlus, C-GRAAL, IsoRank, and Natalie 2.0 with *C. elegans* and *S. cerevisiae* SH3-mediated IINs.

Alignment using BLAST vertex similarity	Greedy	Seed & Extend	GreedyPlus	C-GRAAL	IsoRank	Natalie 2.0
#Represented Protein Orthologies (RPO)	13/16 (81%)	1/16 (6%)	**14/16** (**88%)**	3/16 (19%)	12/16 (75%)	0/16 (0%)
#Orthologous Vertex Pairs (OVP)	**20/22** (**91%)**	1/22 (5%)	18/22 (82%)	4/22 (18%)	19/22 (86%)	0/22 (0%)
#Edges Aligned (EA)	27/466 (6%)	9/466 (2%)	291/466 (62%)	221/466 (47%)	96/466 (21%)	**354/466** (**76%)**

Only BLAST protein scores were used as a similarity feature. The maximum possible values are RPO: 16, OVP: 22, and EA: 466. Bold numbers indicate maximums per column. RPO is the number of known protein orthologies that contain aligned interfaces. OVP is the number of aligned interfaces within orthologous proteins. By definition, OVP ≥ RPO.

**Table 2:** Comparison between GreedyPlus, C-GRAAL, GRAAL, and H-GRAAL with *C. elegans* and *S. cerevisiae* SH3-mediated IINs.

Alignment using graphlet degree vertex similarity	Greedy	Seed & Extend	GreedyPlus	C-GRAAL	GRAAL	H-GRAAL
#Represented Protein Orthologies (RPO)	**1/16** (**6%)**	0/16 (0%)	0/16 (0%)	0/16 (0%)	0/16 (0%)	**1/16** (**6%)**
#Orthologous Vertex Pairs (OVP)	**1/22** (**5%)**	0/22 (0%)	0/22 (0%)	0/22 (0%)	0/22 (0%)	**1/22** (**5%)**
#Edges Aligned (EA)	91/466 (20%)	**319/466** (**68%)**	298/466 (64%)	295/466 (63%)	157/466 (34%)	93/466 (20%)

Only graphlet similarity scores were used as a similarity feature. The maximum possible values are RPO: 16, OVP: 22, and EA: 466. Bold numbers indicate maximums per column. RPO is the number of known protein orthologies that contain aligned interfaces. OVP is the number of aligned interfaces within orthologous proteins. By definition, OVP ≥ RPO.

**Table 3:** Alignment algorithm performance on *C. elegans* and *S. cerevisiae* SH3-mediated IINs using all similarity features.

Alignment using 29 equal weight vertex similarity measures	Greedy	Seed & Extend	GreedyPlus	C-GRAAL	GRAAL	H-GRAAL	IsoRank	Natalie 2.0
**#Represented Protein Orthologies (RPO)**	10/16 (63%)	2/16 (13%)	7/16 (44%)	1/16 (6%)	4/16 (25%)	**11/16** (**69%)**	7/16 (44%)	0/16 (0%)
**#Orthologous Vertex Pairs (OVP)**	13/22 (59%)	2/22 (9%)	10/22 (45%)	1/22 (5%)	5/22 (23%)	**15/22** (**68%)**	9/22 (41%)	0/22 (0%)
**#Edges Aligned (EA)**	35/466 (8%)	305/466 (65%)	238/466 (51%)	293/466 (63%)	56/466 (12%)	47/466 (10%)	87/466 (19%)	**354/466** (**76%)**
**Runtime (ms)**	766	794	2,719	2,755	**2,695**	84,722	112,289	1,804,620*

All 29 similarity features were used with naïve parameterization. The maximum possible values are RPO: 16, OVP: 22, and EA: 466. Bold numbers indicate maximums per column. RPO is the number of known protein orthologies that contain aligned interfaces. OVP is the number of aligned interfaces within orthologous proteins. By definition, OVP ≥ RPO.

^*^The original distribution was used for Natalie 2.0. All other algorithms were implemented in Java by the authors.

**Table 4:** A reduced “optimal” parameter set for GreedyPlus, normalized out of 100.

Proteins			
BLAST score	46.86	TCSS – molecular function	5.71
**Domains**			
BLAST score	1.14	Closeness centrality	20.71
**Ligands**			
Closeness centrality	1.71	Smith-Waterman score	16.00

Edge alignment weight	8.00		

A reduced set of similarity features used by GreedyPlus to achieve “optimal” alignment performance, and their associated weights.

**Table 5:** GreedyPlus performance using different similarity features.

	Represented Protein Orthologies	Orthologous Vertex Pairs	Edges Aligned
**Protein BLAST**	10	12	299
**Domain BLAST / Ligand S-W**	0	0	296
**Protein & Domain BLAST / Ligand S-W**	6	10	288
**Topological Features**	0	0	272
**Functional Features**	5	7	278

GreedyPlus was used to align the *C. elegans* and *S. cerevisiae* SH3 IINs, using different sets of similarity features under simple parameterization (all scoring weights, including EAW, are equal), and the results of each alignment are shown.
