# Supplementary material for: GreedyPlus: An Algorithm for the Alignment of Interface Interaction Networks
Source: Sci Rep. 2015 Jul 13;5:12074. doi: 10.1038/srep12074 (PMC4499810; doi:10.1038/srep12074)
Supplement: Supplementary Information [file srep12074-s1.pdf]

GreedyPlus: An Algorithm for the Alignment of Interface Interaction Networks

Brian Law[1,2], **Gary D. Bader**[1,2,3§]

[1]Department of Computer Science, University of Toronto, Toronto, ON, Canada

[2]The Donnelly Centre, University of Toronto, Toronto, ON, Canada

[3]Department of Molecular Genetics, University of Toronto, Toronto, ON, Canada

[§]Corresponding author

Email addresses:

BL: bm.law@utoronto.ca

GDB: gary.bader@utoronto.ca

**Proteins**			
BLAST coverage	8.48	BLAST score	6.89
TCSS - biological process	0.62	TCSS - cellular component	2.56
TCSS - molecular function	6.98		
**Domains**			
Average shortest path length	7.60	Betweenness centrality	5.12
BLAST coverage	6.71	BLAST score	2.65
Closeness centrality	6.54	Degree	0.53
Eccentricity	1.24	Graphlet degree similarity	7.16
Neighbourhood connectivity	2.74	Radiality	4.42
Stress	7.69	Topological coefficient	1.86
**Ligands**			
Average shortest path length	1.94	Betweenness centrality	4.06
Closeness centrality	0.88	Degree	0.17
Eccentricity	1.50	Graphlet degree similarity	0.27
Neighbourhood connectivity	0.53	Radiality	0.44
Smith-Waterman coverage	5.04	Smith-Waterman score	1.33
Stress	1.50	Topological coefficient	0.71
Edge alignment weight	1.86		

Supplementary Table 1 - An "optimal" parameter set for GreedyPlus, normalized out of 100.

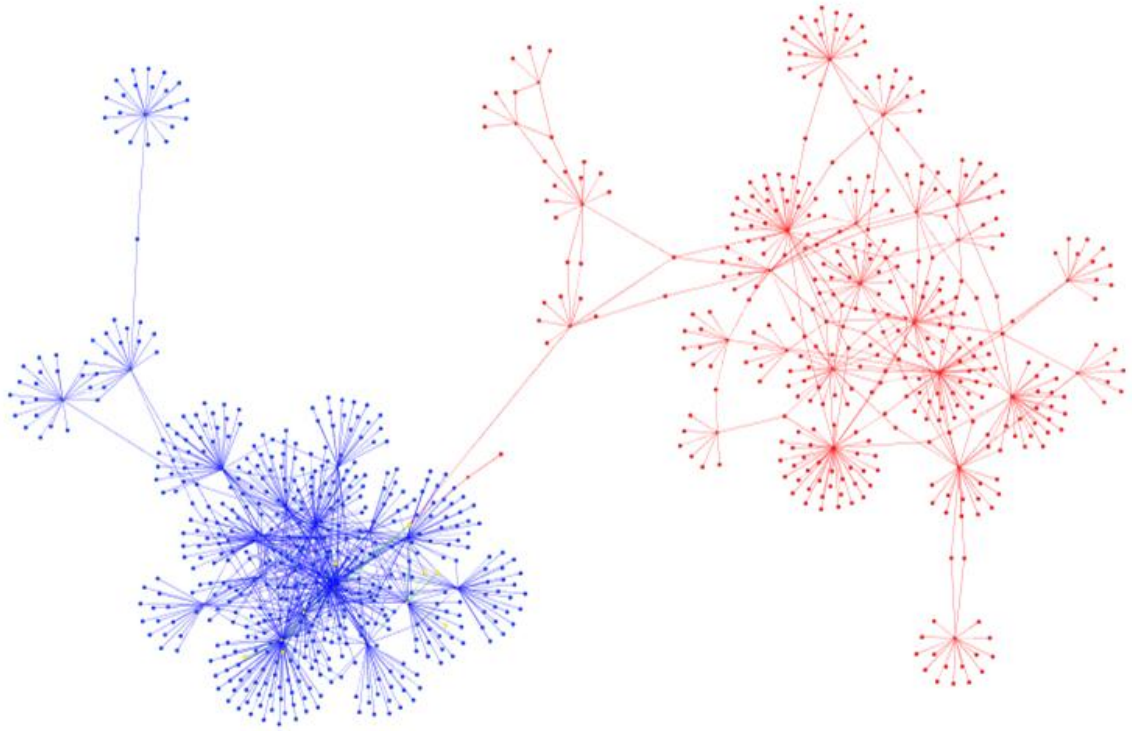

Supplementary Figure 1 - Single seed and extend alignment of worm and yeast SH3-mediated IINs, using protein BLAST as the only similarity feature. Domains are represented by triangular vertices, ligands by circular vertices. Green vertices and edges are aligned, red are unaligned from worm, blue are unaligned from yeast. Very few (10) vertices are aligned due to a poor choice in starting seed position by the algorithm.

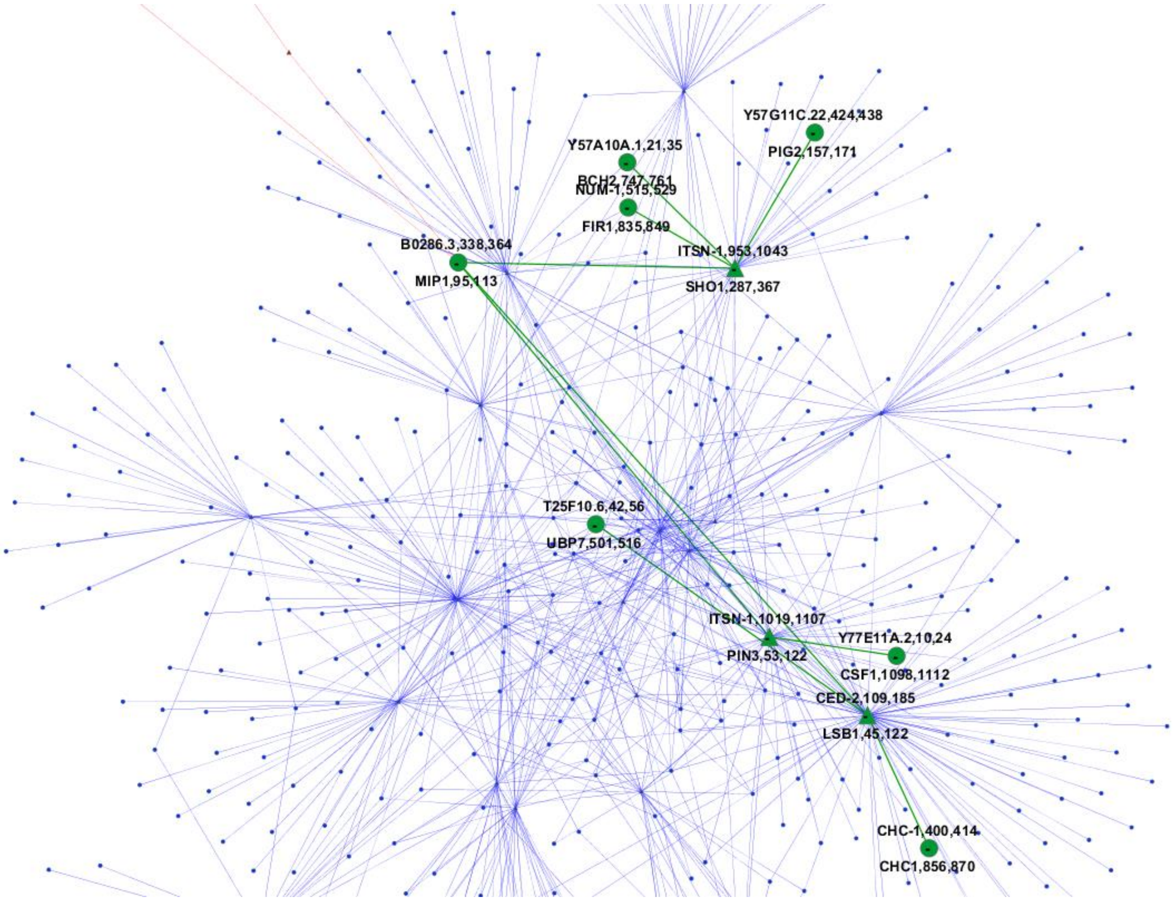

Supplementary Figure 2 - A zoomed-in view of, highlighting the aligned vertices from the alignment. Label notation is of the form {protein name,start position,end position}. Notably, only the aligned vertex B0286.3,338,364 - MIP1,95,113 has any adjacent unaligned yeast-network edges, but it has no more adjacent unaligned worm-network edges to continue alignment.
